# Redefining multiple sclerosis therapy through microbial immunomodulation and epigenetic control

**DOI:** 10.1016/j.jtauto.2025.100313

**Published:** 2025-08-29

**Authors:** Shan Xu, Christina James Thomas, Sunilgowda Sunnagatta Nagaraja, Rakesh Kumar, Kamlesh Sawant, Duminduni Hewa Angappulige, Andy Fang Song, Krish Suman, Benjamin Borja, Paul de Figueiredo, Jianxun Song

**Affiliations:** a Department of Nutrition, Texas A&M University, College Station, TX, 77843, USA; b Department of Microbial Pathogenesis and Immunology, Texas A&M University Health Science Center, MREB II, Room 3344, 8447 John Sharp Parkway, Bryan, TX, 77807, USA; c Department of Molecular Microbiology and Immunology, University of Missouri, Columbia, MO, 65212, USA

**Keywords:** Multiple sclerosis, Immunology, Epigenetics, Neuroinflammation, Microbiome, T cell regulation

## Abstract

Multiple sclerosis (MS) is a chronic autoimmune disorder marked by immune-driven demyelination and neurodegeneration in the central nervous system. This Review explores the immunological, molecular, and epigenetic underpinnings of MS, emphasizing T and B cell involvement, dysregulated signaling pathways (e.g., TGF-β, Akt, Wnt), and the role of cell death in disease progression. Epigenetic mechanisms—such as DNA methylation and histone modifications, further modulate immune responses. While current therapies broadly suppress immunity, emerging approaches, including engineered bacteria, microbiome-based interventions, and cell therapies, offer targeted immune modulation and neuroprotection. Together, these strategies illuminate a path toward next-generation MS treatments with improved precision and efficacy.

## Highlights

• Recent innovations in microbiome-based and bacterial-engineered immunotherapies provide targeted strategies for MS intervention.
• Dysregulated T and B cell signaling plays a pivotal role in neuroinflammation and CNS demyelination.
• Epigenetic mechanisms such as DNA methylation and histone modification modulate immune cell function in MS.
• Future therapies will likely integrate immune modulation, signaling pathway correction, and neuroprotective strategies.

## 1 Immune attacks on the CNS: how MS starts and progresses

Multiple Sclerosis (MS) is a chronic autoimmune disease characterized by inflammation and demyelination within the central nervous system (CNS), ultimately leading to neurodegeneration and progressive disability. Globally, MS affects approximately two million people, with prevalence rates as high as 100,000 in the United Kingdom alone [1]. Typically manifesting between the ages of 20 and 40, MS disproportionately affects women, with a female-to-male ratio of about 3:1 [2]. The chronic nature of the disease, coupled with substantial healthcare costs and personal impact, significantly burdens affected individuals and society [3] (see Table 1, Table 2).

**Table 1:** Key cytokines, Janus kinases (JAKs), and signal transducers (STATs) regulating T cell proliferation, Th1/Th17 differentiation, regulatory T cell maintenance, and macrophage polarization in MS.

Immune Function	Key Cytokines	JAKs Involved	STATs Activated	Cell Type/Subset	Downstream Genes/Pathways	Functional Outcome	Refs
**T Cell Proliferation**	IL-2, IL-7, IL-15	JAK1, JAK3	STAT5A, STAT5B	CD4^+^, CD8^+^ T cells	*BCL2, MYC, Cyclin D3*	Promotes survival, proliferation, and clonal expansion of activated T cells	[28,33]
**Th17 Differentiation**	IL-6, IL-21, IL-23, TGF-β	JAK1, JAK2, TYK2	STAT3	Th17 cells	*RORγt, IL1*7 A/F*, IL21, IL23R*	Drives proinflammatory Th17 cell lineage; critical in MS pathogenesis	[13,37]
**Th1 Differentiation**	IL-12	JAK2, TYK2	STAT4	Th1 cells	*TBX21 (T-bet), IFNG*	Promotes Th1 polarization, IFN-γ production, and CNS inflammation	[13]
**Treg Homeostasis**	IL-2	JAK1, JAK3	STAT5	Regulatory T cells (Tregs)	*FOXP3, CD25, CTLA4*	Supports Treg differentiation and suppressive function; balances effector T cells	[39,40]
**M1 Macrophage Activation**	IFN-γ, LPS	JAK1, JAK2	STAT1	M1 (classically activated)	*iNOS, IL12, TNFA*	Induces proinflammatory macrophage phenotype; promotes neuroinflammation	[35]
**M2 Macrophage Activation**	IL-10, IL-4	JAK1, TYK2 (IL-10); JAK1, JAK3 (IL-4)	STAT3 (IL-10), STAT6 (IL-4)	M2 (alternatively activated)	*ARG1, MRC1 (CD206), IL10*	Induces anti-inflammatory macrophages; promotes resolution of inflammation	[41]
**T Cell Exhaustion & Anergy**	IL-27, IL-10	JAK1, JAK2, TYK2	STAT1, STAT3	Chronic T cells	*PDCD1 (PD-1), LAG3, IL10*	Promotes exhaustion and limits overactivation; may impair CNS viral clearance	[14,35]
**B Cell Maturation**	IL-6, IL-21	JAK1, JAK2	STAT3	B cells, Plasma cells	*BCL6, PRDM1 (BLIMP-1), AID*	Supports B cell differentiation and antibody production in MS lesions	[17]

**Table 2:** Cellular components of adaptive and innate immunity involved in multiple sclerosis pathogenesis, highlighting effector and regulatory mechanisms contributing to autoimmune demyelination, neuroinflammation, and neurodegeneration.

Immune Cell/Concept	Key Features/Molecules	Role in MS Pathology	Refs
CD4^+^ T Cells	Th1 cells produce IFN-γ; Th17 cells produce IL-17	Activate microglia/macrophages; disrupt BBB; promote inflammation	[14]
Regulatory T Cells (Tregs)	FoxP3^+^; may secrete IL-10 or become IL-17^+^ under cytokine pressure	Suppress effector T cells; impaired function in MS leads to inflammation	[40,59]
CD8^+^ T Cells	Release perforin/granzyme, Fas/FasL, IL-17	Kill oligodendrocytes/neurons; promote neurodegeneration	[62]
γδ T Cells	FasL, granzyme B, perforin-mediated cytotoxicity	Found in CSF; contribute to demyelination	[61,63]
MAIT Cells	Produce IL-17; semi-invariant TCR	Promote neuroinflammation; depletion reduces disease severity	[40,63]
Molecular Mimicry	TCR cross-reactivity with microbial and self-antigens	Triggers autoreactive T cells in genetically susceptible individuals	[64,65]
B Cells	Produce autoantibodies, present antigen, secrete cytokines	Promote demyelination, chronic inflammation; oligoclonal bands in CSF	[66]
Microglia	Produce TNF-α, IL-1β, IL-6, ROS, NO	Drive neuroinflammation, impair remyelination	[68]
Macrophages	M1: proinflammatory; M2: repair-associated	M1 predominates in MS, exacerbating demyelination	[41]
Dendritic Cells	Conventional DCs activate Th1/Th17; pDCs tolerogenic	cDCs promote autoimmunity; pDCs impaired in MS	[69]
NK Cells	Pro-inflammatory or regulatory subsets	In MS: fewer regulatory NK cells; heightened inflammation	[70]

Clinically, MS presents a broad spectrum of neurological symptoms reflecting the multifocal involvement of CNS. Common manifestations include sensory disturbances (numbness and tingling), motor dysfunction (weakness and spasticity), and visual disturbances related to optic neuritis or ocular motor abnormalities [4]. Given this variability, MS is classified into several subtypes according to disease course. The most prevalent subtype is Relapsing-Remitting MS (RRMS), marked by episodic neurological relapses followed by periods of partial or complete recovery (remissions) [5]. Over time, most RRMS patients transition to Secondary Progressive MS (SPMS), characterized by a gradual, irreversible decline in neurological function independent of relapses. In about 15 % of patients, the disease initially presents as Primary Progressive MS (PPMS), defined by continuous neurological worsening from disease onset without clear relapses [6]. Additionally, some individuals initially present with Clinically Isolated Syndrome (CIS), a first episode of neurological symptoms suggestive of demyelination, which may or may not evolve into definitive MS based on subsequent clinical and radiological findings [7].

## 2 Pathogenesis of multiple sclerosis

MS arises from a complex interplay of genetic, environmental, and immunological factors that collectively drive chronic autoimmune-mediated injury to the central nervous system (CNS) [8]. Genome-wide association studies have identified over 200 genetic loci linked to MS susceptibility, implicating genes involved in T cell activation, antigen presentation, and immune regulation [9]. These findings highlight the central role of immune dysregulation in MS pathogenesis. Environmental factors, such as vitamin D deficiency, Epstein-Barr virus infection, cigarette smoking, and high dietary salt intake, amplify disease risk by exacerbating immune dysfunction and breaking down peripheral tolerance, both hallmarks of autoimmunity [10].

At the core of MS pathophysiology is an aberrant immune response targeting the myelin sheath, the insulating layer around axons essential for neuronal signaling [10]. Autoreactive T cells and pathogenic B cell responses mediate a chronic inflammatory state that leads to demyelination and axonal injury. A key initiating event disrupts the blood-brain barrier (BBB), driven by proinflammatory cytokines and chemokines that activate endothelial cells and compromise BBB integrity [11]. This breach allows autoreactive T cells and other immune effectors to infiltrate the CNS, triggering inflammation and tissue damage [12].

Among the immune cell subsets involved, CD4^+^ T helper (Th) cells—particularly Th1 and Th17 subsets—play pivotal roles [13]. Th1 cells produce interferon-gamma (IFN-γ), which activates macrophages and microglia to release proinflammatory mediators, amplifying demyelination and neuronal damage [14]. Th17 cells secrete interleukin-17 (IL-17), which disrupts the BBB and promotes the infiltration of neutrophils and additional immune cells, further escalating inflammation and lesion formation [15]. These processes create a self-amplifying inflammatory loop within the CNS, emphasizing the central role of autoreactive T cells in MS pathogenesis and identifying them as key therapeutic targets [16].

B cells also play significant roles by presenting antigens, secreting proinflammatory cytokines, and producing myelin-specific autoantibodies, thereby sustaining inflammation and tissue injury [17]. Histopathological studies have revealed ectopic lymphoid-like aggregates in the meninges of MS patients, particularly in progressive forms of the disease. These structures are associated with increased cortical demyelination and correlate with more severe disease progression [18].

Beyond sustaining inflammation, immune cells contribute directly to neurodegeneration, illustrating how chronic immune activation and neurodegeneration are interdependent processes shaping the course of the disease [11]. Persistent inflammation within the CNS leads to oxidative stress, mitochondrial dysfunction, and excitotoxicity, culminating in irreversible axonal injury and neuronal death [19]. Reactive astrocytes respond by forming glial scars that inhibit remyelination and neuronal repair, further worsening neurodegeneration [20].

This persistent cycle of inflammation and neurodegeneration impairs the brain's capacity for repair. It contributes to the diverse and progressive neurological symptoms experienced by MS patients, including motor dysfunction, sensory disturbances, visual impairment, fatigue, and cognitive decline [21]. While inflammation predominates in the early stages of disease, neurodegeneration becomes the principal driver of long-term disability in progressive forms of MS [22]. These insights underscore the need for therapies beyond immunosuppression, aiming instead to promote remyelination, neuroprotection, and CNS repair [23].

Emerging evidence suggests that MS shares overlapping pathological mechanisms with other neurological disorders, such as Alzheimer's disease, Parkinson's disease, and glioblastoma, highlighting opportunities for comparative research and therapeutic innovation [24,25]. Dissecting the cell signaling and molecular pathways that govern immune dysfunction, CNS infiltration, and neurodegeneration are crucial for developing more effective and comprehensive treatments [26].

## 3 Cell signaling and molecular mechanism of MS

**Fig. 1:**
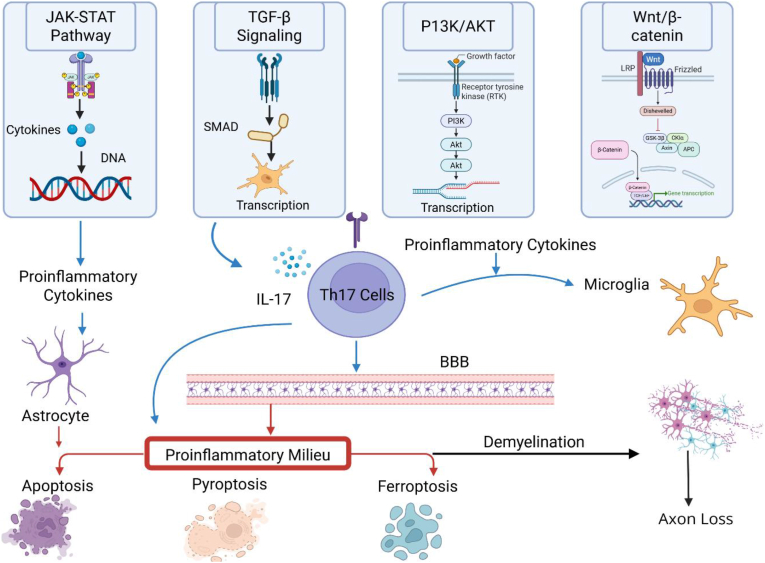
Cytokine Signaling and Immune-Mediated Mechanisms in MS Pathogenesis. JAK/STAT, TGF-β, PI3K/AKT, and Wnt/β-catenin signaling pathways promote Th17 differentiation and IL-17 secretion. IL-17 disrupts the blood-brain barrier (BBB), activates astrocytes and microglia, and establishes a proinflammatory milieu in the CNS. This environment induces multiple forms of regulated cell death, including apoptosis, pyroptosis, and ferroptosis, contributing to demyelination and subsequent axonal loss—key features of neurodegeneration in MS. The figure was created with BioRender (biorender.com).

Building on the immunopathological framework of MS, it is crucial to delineate the intracellular signaling pathways that coordinate immune cell behavior, CNS infiltration, and neurodegeneration [27]. Several dysregulated pathways, including the Janus kinase/signal transducer and activator of transcription (JAK/STAT) axis [28], immunoregulatory signals like transforming growth factor-beta (TGF-β) [29], and homeostatic regulators such as the PI3K/AKT and Wnt/β-catenin pathways, collectively shape the proinflammatory environment and modulate the balance between effector and regulatory immune responses [30,31]. These pathways govern T cell polarization and cytokine production, impact BBB integrity, and influence glial activation and neuronal survival. In addition, abnormal activation of cell death pathways, such as apoptosis, pyroptosis, and ferroptosis, exacerbates oligodendrocyte loss and axonal degeneration, thus accelerating disease progression (Fig. 1).

### 3.1 JAK/STAT pathway and Th17 polarization

The JAK/STAT pathway regulates immune responses and inflammation processes intimately linked to MS development [28]. Dysregulation of this signaling cascade has been associated with various autoimmune and chronic inflammatory diseases [32]. In MS, aberrant JAK/STAT signaling drives peripheral immune cell activation and central neuroinflammation [33]. Proinflammatory cytokines—such as interleukin-6 (IL-6), IL-23, IL-12, and type I/II interferons-are potent activators of this pathway [34]. These cytokines engage JAK family kinases (JAK1, JAK2, JAK3, TYK2), which phosphorylate and activate STAT proteins (particularly STAT1, STAT3, and STAT4), leading to transcription of genes involved in immune cell proliferation and polarization [35].

Among these proteins, STAT3 is particularly critical for Th17 cell differentiation. Its activation by IL-6 and IL-23 induces RORγt, the master transcription factor for Th17 lineage commitment [36]. The resulting expansion of Th17 cells leads to elevated production of IL-17, a proinflammatory cytokine that sustains CNS inflammation and disrupts the BBB, facilitating further immune cell infiltration [37,38].

### 3.2 NF-κB signaling

The NF-κB pathway is also pivotal in orchestrating immune responses, inflammation, and cell survival [42]. Activated by stimuli such as infections, cellular stress, and cytokines, this pathway has been linked to cancer, chronic inflammation, and autoimmune diseases [43]. In MS, NF-κB signaling in peripheral immune cells and CNS-resident glia drives neuroinflammation and contributes to demyelination and neuronal damage [44].

### 3.3 PI3K/AKT/mTOR pathway

The PI3K/AKT/mTOR (PAM) pathway integrates signals from growth factors, metabolic cues, and stress to regulate diverse cellular functions, including growth, survival, and metabolism [35,45]. In the immune system, this pathway orchestrates T cell activation and survival, balancing effector and regulatory T cell (Treg) responses. It also governs B cell maturation, antibody production, and macrophage polarization (proinflammatory M1 vs. anti-inflammatory M2) [46]. PI3K, a lipid kinase complex, generates phosphatidylinositol 3,4,5-trisphosphate (PIP3), which recruits proteins like AKT and PDK1. AKT is then fully activated through phosphorylation by PDK1 and mTOR complex 2 (mTORC2). This cascade shapes immune cell fate and modulates upstream pathways, including ERK and cytokine receptors, thereby influencing JAK/STAT signaling [47].

The mTOR network itself plays a prominent role in MS. Inhibitors of mTOR, such as rapamycin, have shown promise in experimental autoimmune encephalomyelitis (EAE), the animal model of MS [48]. These agents protect neurons and glial cells from oxidative stress-induced injury and promote Treg expansion while dampening pathogenic T cell activity [49]. However, evidence suggests that targeting the PI3K/mTOR axis alone may not drive remyelination in progressive MS [49,50].

These insights underscore the importance of the PI3K/AKT/mTOR axis and other interconnected pathways in regulating immune cell differentiation, survival, and neuroprotection in MS [51]. Given their roles in both adaptive and innate immunity, these signaling pathways remain promising therapeutic targets for modulating inflammation and supporting CNS repair [52]. A deeper understanding of these molecular networks will be essential for developing next-generation treatments that curb autoimmunity and promote neurodegeneration and functional recovery in MS.

## 4 Immune dysregulation in MS: roles of adaptive and innate immunity

**Fig. 2:**
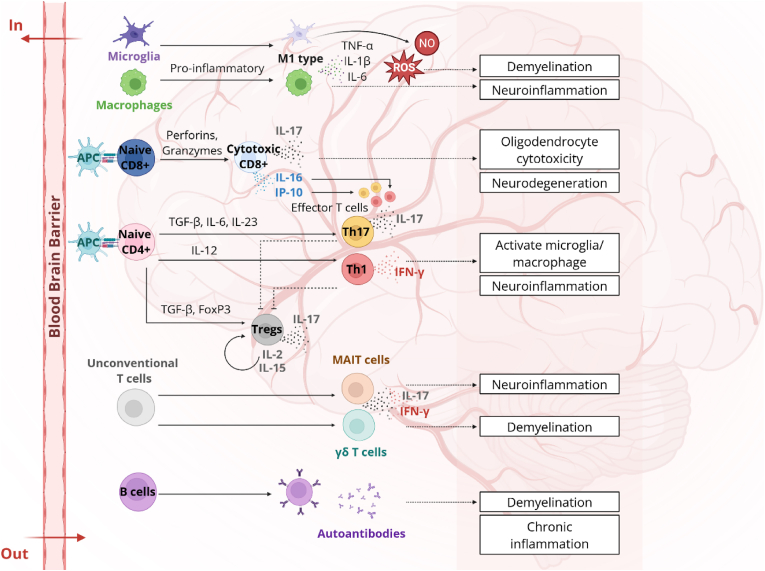
Immunopathogenesis of MS. This schematic illustrates the dynamic interplay between adaptive and innate immunity in MS pathogenesis, highlighting the directional migration of immune cells across the blood-brain barrier (BBB). Arrows labeled “In” indicate the infiltration of autoreactive CD4^+^ T helper cells (Th1 and Th17) and CD8^+^ cytotoxic T cells from the periphery into the CNS. Once within the CNS, Th1 and Th17 cells produce proinflammatory cytokines (IFN-γ and IL-17) that activate microglia and macrophages, further amplifying local inflammation. B cells similarly migrate across the BBB, producing myelin-specific autoantibodies and forming ectopic follicles that perpetuate inflammation and tissue injury. Innate immune cells, including resident microglia and infiltrating macrophages, release TNF-α, IL-6, and reactive oxygen and nitrogen species, driving demyelination and neuronal degeneration. While immune cell egress (“Out”) from the CNS is limited in MS, this imbalance reinforces chronic neuroinflammation. Figure created with BioRender (biorender.com).

Central to MS pathogenesis is the interplay between adaptive and innate immunity [53]. While the adaptive immune system drives antigen-specific attacks on CNS components, the innate immune system amplifies inflammation. It shapes the autoimmune environment, contributing to the disease's chronic and relapsing course [54]. These immune networks collectively orchestrate demyelination, axonal injury, and neurodegeneration (Fig. 2). Understanding this immune dysregulation's cellular and molecular mechanisms is essential for developing targeted immunotherapies [10].

### 4.1 Adaptive immunity in MS

The adaptive immune system, designed to recognize and respond to specific antigens, plays a pivotal role in MS. Dysregulation of adaptive immunity results in an autoimmune attack against CNS components, particularly myelin antigens [54]. Key contributors include autoreactive CD4^+^ and CD8^+^ T cells, B cells, proinflammatory cytokines, and autoantibodies, all of which sustain chronic neuroinflammation and tissue damage [55].

CD4^+^ T helper (Th) cells, especially the Th1 and Th17 subsets, are central to initiating and sustaining CNS inflammation [56]. Th1 cells, activated by IL-12, express T-bet and produce IFN-γ, which activates microglia and macrophages. Th17 cells, induced by TGF-β, IL-6, and IL-23, express RORγt and secrete IL-17, disrupting the BBB and recruiting additional immune cells [57]. Genetic factors, such as HLA-DRB1∗15:01, enhance autoreactive T cell responses and increase MS susceptibility [8]. Notably, myelin-reactive CD4^+^ T cells are found at elevated frequencies in MS patients, reflecting a failure of peripheral tolerance [58].

Regulatory T cells (Tregs), marked by FOXP3 expression, are crucial for maintaining immune homeostasis [59]. In MS, Tregs are often reduced or functionally impaired, leading to unchecked effector T cell activity. Environmental cytokines, such as IL-2 and IL-15, can even reprogram Tregs to produce IL-17, further fueling inflammation. This Treg dysfunction is a critical failure of immune regulation [60].

CD8^+^ cytotoxic T cells directly contribute to neuronal and oligodendrocyte injury by releasing perforin and granzyme B and engaging Fas/FasL pathways [61]. Their presence in MS lesions underscores their role in both acute demyelination and chronic neurodegeneration [62]. Unconventional T cell subsets, including γδ T cells and mucosal-associated invariant T (MAIT) cells, have also emerged as significant contributors. γδ T cells, enriched in the cerebrospinal fluid (CSF) of MS patients, exhibit cytotoxic activity through FasL, granzyme B, and perforin [63]. MAIT cells, which are increased in MS brain tissue, produce IL-17 and amplify neuroinflammation. Their depletion in animal models reduces disease severity, highlighting their pathogenic potential [40].

MS pathogenesis is further driven by molecular mimicry, in which T cells activated by microbial antigens cross-react with myelin proteins [64]. T cell receptor (TCR) degeneracy, whereby a single TCR recognizes multiple antigens, also broadens autoreactivity. These mechanisms suggest that infections or microbiota-derived antigens may trigger autoimmunity in genetically predisposed individuals [65].

B cells contribute multifaceted roles in MS, acting as antigen-presenting cells, producing proinflammatory cytokines, and generating myelin-specific autoantibodies that drive complement-mediated injury [66]. The presence of oligoclonal IgG bands in CSF—an MS hallmark—reflects chronic B-cell activation within the CNS.

### 4.2 Innate immunity in MS

Although adaptive immunity initiates the autoimmune cascade, innate immune cells are fundamental in sustaining inflammation and shaping adaptive responses [67]. Microglia, infiltrating macrophages, dendritic cells (DCs), and natural killer (NK) cells are all implicated through persistent activation and failure to resolve inflammation [10,68].

Microglia, the resident immune cells of the CNS, adopted a pro-inflammatory M1-like phenotype early in MS, releasing TNF-α, IL-1β, IL-6, and reactive oxygen and nitrogen species that damage myelin and neurons [68]. Chronic microglial activation also impairs remyelination by hindering myelin debris clearance. Infiltrating macrophages similarly exhibit an M1-like phenotype in MS, driving pro-inflammatory cytokine production and exacerbating demyelination, while M2-like macrophages, which promote repair, are less effective [41].

DCs are key antigen-presenting cells that prime adaptive immune responses. In MS, conventional DCs promote Th1/Th17 polarization, while plasmacytoid DCs, typically tolerogenic, are impaired, limiting their ability to control inflammation [69]. NK cells display dual roles: proinflammatory NK subsets enhance inflammation, while regulatory NK cells, which suppress T cell activity, are diminished in MS, contributing to disease progression [70].

## 5 Epigenetics of MS

**Fig. 3:**
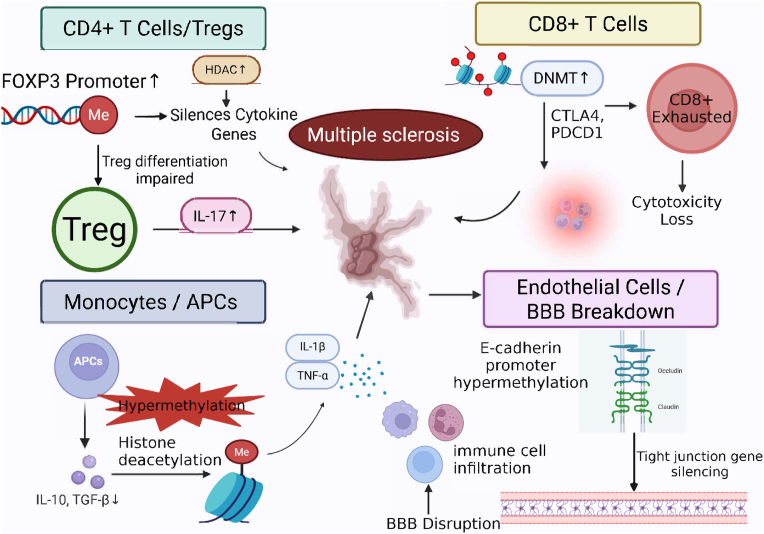
Epigenetic Regulation of T Cell Fate and Immune Dysfunction MS. This figure illustrates how cell type, specific epigenetic modifications disrupt immune homeostasis in MS. In CD4^+^ T cells, DNA hypermethylation at the *FOXP3* promoter and increased histone deacetylase (HDAC) activity impair regulatory T cell (Treg) differentiation and cytokine gene expression, promoting a proinflammatory shift (e.g., IL-17). CD8^+^ T cells exhibit DNA methylation, mediated silencing of immune checkpoint genes (*CTLA4*, *PDCD1*), leading to functional exhaustion and loss of cytotoxicity. In monocytes and antigen-presenting cells (APCs), hypermethylation and histone modifications suppress tolerogenic cytokines (e.g., IL-10, TGF-β) and enhance proinflammatory responses (e.g., IL-1β, TNF-α). In endothelial cells, hypermethylation of tight junction genes (e.g., *E-cadherin*) contributes to BBB disruption and immune cell infiltration. Collectively, these epigenetic alterations exacerbate CNS inflammation and MS pathogenesis. Figure created with BioRender (biorender.com).

Recent advances have highlighted the crucial role of epigenetic modifications in influencing phenotypic traits and the pathogenesis of complex diseases, particularly autoimmune and neurocognitive disorders. Epigenetics refers to heritable changes in gene expression that occur without altering the DNA sequence, shaping cellular activity and contributing to disease susceptibility [71]. These modifications include DNA methylation, histone modifications, and regulation by non-coding RNAs (Fig. 3).

### 5.1 DNA methylation

DNA methylation involves adding methyl groups to cytosine-phosphate-guanine (CpG) dinucleotides, catalyzed by DNA methyltransferases (DNMTs). Aberrant methylation patterns have been implicated in neurological and autoimmune disorders, including MS [72]. In MS, immune cells—particularly T cells—show abnormal methylation, contributing to immune dysregulation [73]. For example, irregular methylation of the IFN-γ promoter region in Th1 cells reflects their dominance in MS pathology [74]. In RRMS patients, increased methylation of the E-cadherin promoter in the BBB promotes lymphocyte infiltration into the CNS [74]. Genome-wide methylation studies in B cells from RRMS patients reveal further links to heightened immune activation and inflammatory responses [73]. Notably, demethylation of FOXP3 in regulatory T cells (Tregs) enhances their suppressive function, which is crucial for modulating Th1/Th2 balance [75]. Other studies have identified hypo- and hypermethylated regions in MS patient-derived CD4^+^ T lymphocytes and CD14^+^ monocytes [76]. Genetic variations, such as SNPs within the anti-Müllerian hormone (AMH) gene, have also been linked to methylation changes and disease progression [77]. Furthermore, hypomethylation in B cells increases ATXN1 expression, a mechanistic contributor to MS pathogenesis [78]. Nutritional factors like vitamin D and DNMT inhibitors have shown promise in preclinical models by mitigating harmful hypermethylation of immune-related genes [79]. Although these findings underscore the significance of DNA methylation in MS progression, further studies are needed to establish causality and therapeutic potential.

### 5.2 Histone modifications

Histone modifications also play a crucial role in MS. Histone deacetylases (HDACs) remove acetyl groups from histones, leading to chromatin condensation and transcriptional repression, thereby sustaining pathogenic immune responses [80]. HDAC inhibitors (HDACi) have effectively shifted immune cell profiles from proinflammatory Th1/Th17 to anti-inflammatory Th2/Treg states, reducing autoimmune responses [81]. In EAE models, HDAC11 knockout limits monocyte and dendritic cell infiltration, reducing CNS demyelination and disease severity [82]. Additionally, propionic and butyric acid treatments inhibit class I/II HDACs, enhancing neurons' antioxidant activity and ATP synthesis [83]. HDAC1's role in suppressing HLA-DR expression suggests a link to neurodegeneration in MS [84]. Furthermore, histone methyltransferases (HMTs) regulate gene expression based on the methylation site and degree. Elevated H3K27me3 is associated with suppression of autoimmune responses via tolerogenic dendritic cell regulation, while reduced H3K4me3—a mark of mitochondrial and energy metabolism genes—is linked to axonal degeneration in MS [85]. These epigenetic alterations highlight histone-modifying enzymes as promising therapeutic targets. Continued preclinical research is essential to translate these findings into clinical applications, aiming to modulate immune and neurodegenerative pathways in MS.

## 6 Immunotherapy for MS

MS is a chronic autoimmune disease of the CNS, driven by autoreactive T cells that mistakenly target myelin proteins and trigger progressive neurological damage [7]. Immunotherapy has become a cornerstone of MS management, aiming to modulate immune responses, reduce relapse rates, and slow disease progression [86].

### 6.1 Conventional immunotherapies

Disease-modifying therapies (DMTs) form the backbone of MS treatment. First-line agents include interferon-beta (IFN-β), which modulates immune activity, and glatiramer acetate, which induces immune tolerance by mimicking myelin basic protein [87]. Monoclonal antibodies (mAbs) represent a significant advance in targeted immunomodulation. Natalizumab blocks α4-integrin, preventing immune cell migration into the CNS, while ocrelizumab depletes CD20^+^ B cells, reducing immune-mediated injury [87,88]. Alemtuzumab, targeting CD52^+^ cells, promotes immune system reconstitution [66,89]. Sphingosine-1-phosphate (S1P) receptor modulators such as fingolimod and siponimod sequester lymphocytes within lymph nodes, limiting CNS infiltration [90].

Despite their efficacy in reducing inflammation, these treatments are limited by partial efficacy in progressive disease, risks of opportunistic infections and malignancy, and a lack of mechanisms for CNS repair. Consequently, the need for therapies that restore immune tolerance, support remyelination, and limit neurodegeneration has driven the exploration of innovative approaches.

### 6.2 Emerging and experimental therapies

Novel strategies include chimeric antigen receptor (CAR) T cell therapies designed to selectively eliminate autoreactive B cells, tolerogenic dendritic cells engineered to restore immune tolerance, and regulatory T cell (Treg) therapies aimed at enhancing immune balance in MS patients [91,92].

### 6.3 Bacterial-based immunotherapy

Bacterial-based immunotherapy has emerged as a promising avenue to complement conventional DMTs [93]. Advances in synthetic biology have enabled engineering of bacteria as customizable platforms to deliver immunomodulatory molecules and modulate immune responses [94]. This approach leverages the crucial role of gut microbiota in immune regulation and neuroimmune interactions. Dysbiosis, or microbial imbalance, has been increasingly implicated in MS pathogenesis, leading to proinflammatory responses and impaired immune tolerance [95]. Targeting this dysbiosis through specific bacterial strain such as probiotics, engineered bacteria, or metabolic modulators—represents a novel therapeutic strategy to restore immune homeostasis and reduce neuroinflammation [96,97].

### 6.4 Probiotic and commensal bacteria

Several probiotic strains have demonstrated immunoregulatory effects relevant to MS. *Lactobacillus* species (e.g., *L. reuteri*, *L. plantarum*) enhance gut barrier function, produce short-chain fatty acids (SCFAs) like butyrate, and promote Treg differentiation, dampening pathogenic Th17 activity [39]. *Bifidobacterium* species produce metabolites influencing peripheral and CNS immunity, enhancing anti-inflammatory cytokines while reducing proinflammatory mediators [98,99]. *Faecalibacterium prausnitzii* and *Akkermansia muciniphila* maintain gut homeostasis and have been linked to increased Treg activity and reduced Th1/Th17 responses [100,101]. *Prevotella histicola* has protective effects in EAE models, reducing neuroinflammation through Treg induction [102].

### 6.5 Engineered bacterial strains

**Fig. 4:**
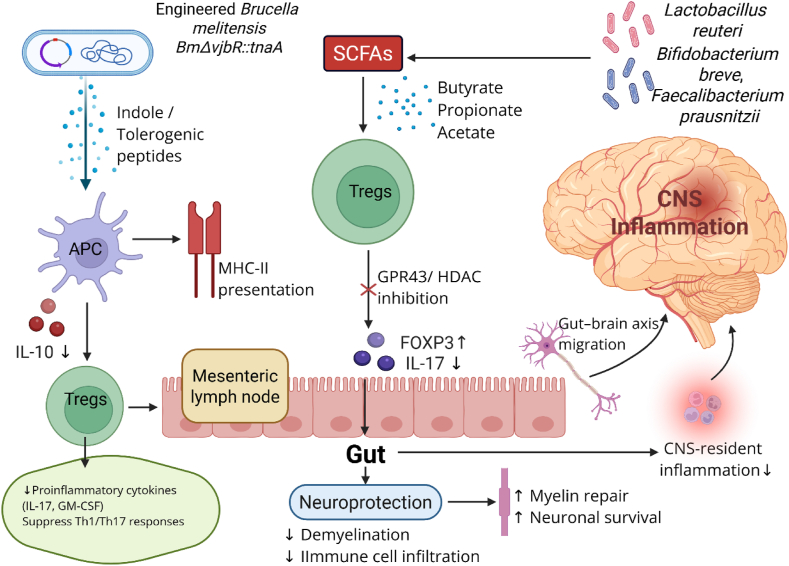
Microbial Immunotherapy and Gut–Brain Axis in MS. This diagram illustrates how engineered and commensal microbes regulate CNS inflammation through gut immune modulation. Engineered *Brucella melitensis* BmΔvjbRtnaA delivers indole and tolerogenic peptides to antigen-presenting cells (APCs), promoting IL-10 production and MHC-II-mediated presentation, which induce FOXP3^+^ regulatory T cells (Tregs) via the mesenteric lymph node. In parallel, commensal bacteria (*Lactobacillus reuteri*, *Bifidobacterium breve*, *Faecalibacterium prausnitzii*) generate short-chain fatty acids (SCFAs; butyrate, propionate, acetate) that enhance Treg differentiation via GPR43 signaling and HDAC inhibition. These Tregs suppress proinflammatory cytokines (e.g., IL-17, GM-CSF), downregulate CNS-resident inflammation, and migrate through the gut–brain axis to promote neuroprotection by reducing demyelination, limiting immune cell infiltration, and supporting myelin repair and neuronal survival. Figure created with BioRender (biorender.com).

Certain facultative intracellular bacteria and genetically modified strains have demonstrated potential in modulating immune responses in MS. Engineered *Brucella melitensis* strains modulate antigen-presenting cell function and promote Treg differentiation, showing promise in restoring immune tolerance [103] (Fig. 4). *Mycobacterium avium* subspecies paratuberculosis (MAP) and genetically modified and has been investigated for its immunoregulatory potential, particularly in modulating Th1/Th17-driven inflammation [104]. *Salmonella enterica* have also been investigated for their ability to induce tolerogenic immune responses [105,106]. *Helicobacter hepaticus* has demonstrated neuroprotective properties by shifting immune profiles toward anti-inflammatory phenotypes [107].

Further, metabolically engineered bacterial strains such as *Lactococcus lactis* expressing myelin autoantigens have shown efficacy in inducing antigen-specific tolerance and reducing CNS inflammation in preclinical models [108]. Other strains, like *Escherichia coli* Nissle 1917 and particular *Clostridium* species, also show promise in modulating immune responses through SCFA production and Treg expansion [109].

Bacterial-based immunotherapies offer the potential for long-term immune tolerance, precise modulation of neuroinflammatory pathways, and reduced systemic immunosuppression compared to conventional drugs [110]. However, challenges remain, including ensuring the safety and stability of bacterial formulations, optimizing delivery mechanisms, and addressing variability in host microbiome responses [111].

## 7 Current treatments and their limitations

The current therapeutic landscape for MS centers on immunomodulatory and, to a lesser extent, immunosuppressive agents. These therapies primarily aim to reduce relapse frequency and delay disease progression [112]. First-line treatments, such as interferon-β and glatiramer acetate, modulate T cell activity to exert anti-inflammatory effects [113]. While generally well-tolerated, these therapies have limited efficacy in preventing long-term disability, as they predominantly target early immune activation and do not adequately address neurodegeneration.

Second-line therapies, including monoclonal antibodies (mAbs) and small-molecule agents, have improved the control of disease activity [114]. Natalizumab blocks α4-integrin to prevent immune cell migration into the CNS, while ocrelizumab depletes CD20^+^ B cells to reduce inflammation and relapses [88]. Fingolimod, an S1P receptor modulator, sequesters lymphocytes within lymph nodes, restricting their CNS entry [115]. Although these treatments offer stronger suppression of immune activity, they have significant limitations.

A key limitation is partial efficacy. These therapies can suppress inflammation and reduce relapse rates but do not entirely halt neurodegenerative processes, leaving patients, especially those with progressive MS, vulnerable to continued decline [116]. Moreover, systemic immunosuppression associated with these agents raises concerns about opportunistic infections (e.g., progressive multifocal leukoencephalopathy caused by the JC virus) and increased malignancy risk [117].

Notably, most current treatments lack mechanisms for CNS repair, failing to promote axonal regeneration, remyelination, or reversal of glial scarring [118]. Reactive astrocytes and chronic neuroinflammation create a non-permissive environment for repair, further limiting the efficacy of these therapies [119].

These shortcomings underscore the need for therapies that regulate immune responses and enable tissue repair and functional recovery. Future treatments should integrate immune modulation with neuroprotection and remyelination strategies to better address the complex pathophysiology of MS [120].

## 8 Concluding remarks and future perspectives

Despite substantial progress, key challenges persist in achieving long-term immune tolerance, effective CNS repair, and minimizing systemic side effects in MS treatment. Future strategies must integrate immune modulation with neuroprotection and regeneration to address the disease's multifaceted pathophysiology.

Emerging approaches—such as bacterial-based immunotherapy, metabolic reprogramming, and epigenetic modulations show promise. For example, engineered bacteria like *Brucella melitensis* strains secreting immunomodulatory molecules could reshape the inflammatory environment and promote regulatory immunity. However, refining bacterial delivery vehicles for safety and optimizing microbiome interactions remain crucial next steps.

Advances in single-cell sequencing and spatial transcriptomics will further illuminate immune cell heterogeneity and CNS-resident immune responses in MS, paving the way for more personalized treatments. Combined with immunotherapies, these insights could enable tailored regimens based on each patient's immunological and molecular profile.

Moreover, therapies aimed at enhancing remyelination, axonal repair, and overcoming the inhibitory CNS microenvironment are priorities. Integrating regenerative strategies—such as stem cell-based or glial scar-modulating therapies—with immunomodulation may shift the treatment focus from mere suppression to proper functional recovery.

Ultimately, a multifaceted therapeutic approach that combines immunology, microbiology, and regenerative medicine holds promise for achieving long-term remission and meaningful neurological recovery in MS.

**Outstanding questions box**

• What are the precise molecular triggers that reprogram autoreactive T cells from peripheral immunosurveillance to pathogenic CNS infiltration and effector function?
• How do distinct B cell subsets balance neurodegenerative versus reparative roles within progressive forms of multiple sclerosis?
• What cellular and molecular factors govern the transition from acute inflammatory demyelination to chronic neurodegenerative pathology in MS, and how can this inform therapeutic interventions?
• Can engineered bacterial-based immunotherapies be harnessed to induce durable immune tolerance while preserving gut-CNS homeostasis and minimizing off-target effects?
• In what ways does the CNS microenvironment—including reactive glia and extracellular matrix remodeling—shape immune cell trafficking, phenotype, and effector function in MS lesions?
• To what extent do epigenetic modifications in CNS-resident versus peripheral immune cell populations orchestrate disease progression and response to therapeutic intervention?
• How might the integration of spatial transcriptomics and single-cell multi-omics approaches refine our understanding of cellular heterogeneity and functional states driving MS pathogenesis?
• What are the mechanistic commonalities and therapeutic opportunities shared between MS and other neuroinflammatory or neurodegenerative disorders, such as Alzheimer's disease, Parkinson's disease, and glioblastoma?

## CRediT authorship contribution statement

**Shan Xu:** Writing – original draft. **Christina James Thomas:** Validation. **Sunilgowda Sunnagatta Nagaraja:** Resources. **Rakesh Kumar:** Methodology. **Kamlesh Sawant:** Data curation. **Duminduni Hewa Angappulige:** Formal analysis. **Andy Fang Song:** Visualization. **Krish Suman:** Methodology. **Benjamin Borja:** Resources. **Paul de Figueiredo:** Writing – review & editing. **Jianxun Song:** Conceptualization, Funding acquisition, Writing – review & editing.

## Declaration of competing interest

The authors declare the following financial interests/personal relationships which may be considered as potential competing interests:Jianxun Song reports financial support was provided by Texas A&M University. Jianxun Song reports a relationship with Texas A&M University that includes: employment. If there are other authors, they declare that they have no known competing financial interests or personal relationships that could have appeared to influence the work reported in this paper.

## Data Availability

No data was used for the research described in the article.

## Abbreviations

BBB: Blood-Brain Barrier
Bregs: Regulatory B Cells
CAR T cells: Chimeric Antigen Receptor T Cells
CD: Cluster of Differentiation
CNS: Central Nervous System
CpG: Cytosine-Phosphate-Guanine
DCs: Dendritic Cells
DMTs: Disease-Modifying Therapies
DNMTs: DNA Methyltransferases
EAE: Experimental Autoimmune Encephalomyelitis
FOXP3: Forkhead Box P3
GABA: Gamma-Aminobutyric Acid
HDACs: Histone Deacetylases
HLA: Human Leukocyte Antigen
IFN-γ: Interferon Gamma
IL: Interleukin
IP-10: Interferon Gamma-Induced Protein 10
JAK/STAT: Janus Kinase/Signal Transducer and Activator of Transcription
MAIT cells: Mucosal-Associated Invariant T Cells
MAP: *Mycobacterium avium* subspecies paratuberculosis
miRNA: MicroRNA
MS: Multiple Sclerosis
mAbs: Monoclonal Antibodies
NK cells: Natural Killer Cells
NO: Nitric Oxide
pDCs: Plasmacytoid Dendritic Cells
PPARγ: Peroxisome Proliferator-Activated Receptor Gamma
RA: Rheumatoid Arthritis
RORγt: RAR-Related Orphan Receptor Gamma t
ROS: Reactive Oxygen Species
RRMS: Relapsing-Remitting Multiple Sclerosis
S1P: Sphingosine-1-Phosphate
SCFAs: Short-Chain Fatty Acids
SNPs: Single Nucleotide Polymorphisms
T-bet: T-box Transcription Factor TBX21
TCR: T Cell Receptor
TGF-β: Transforming Growth Factor Beta
Th cells: T Helper Cells
Tregs: Regulatory T Cells
TYK2: Tyrosine Kinase 2
